# Supplementary figures and images for: Sequencing and analyses on chloroplast genomes of Tetrataenium candicans and two allies give new insights on structural variants, DNA barcoding and phylogeny in Apiaceae subfamily Apioideae
Source: PeerJ. 2019 Nov 21;7:e8063. doi: 10.7717/peerj.8063 (PMC6875388; doi:10.7717/peerj.8063)

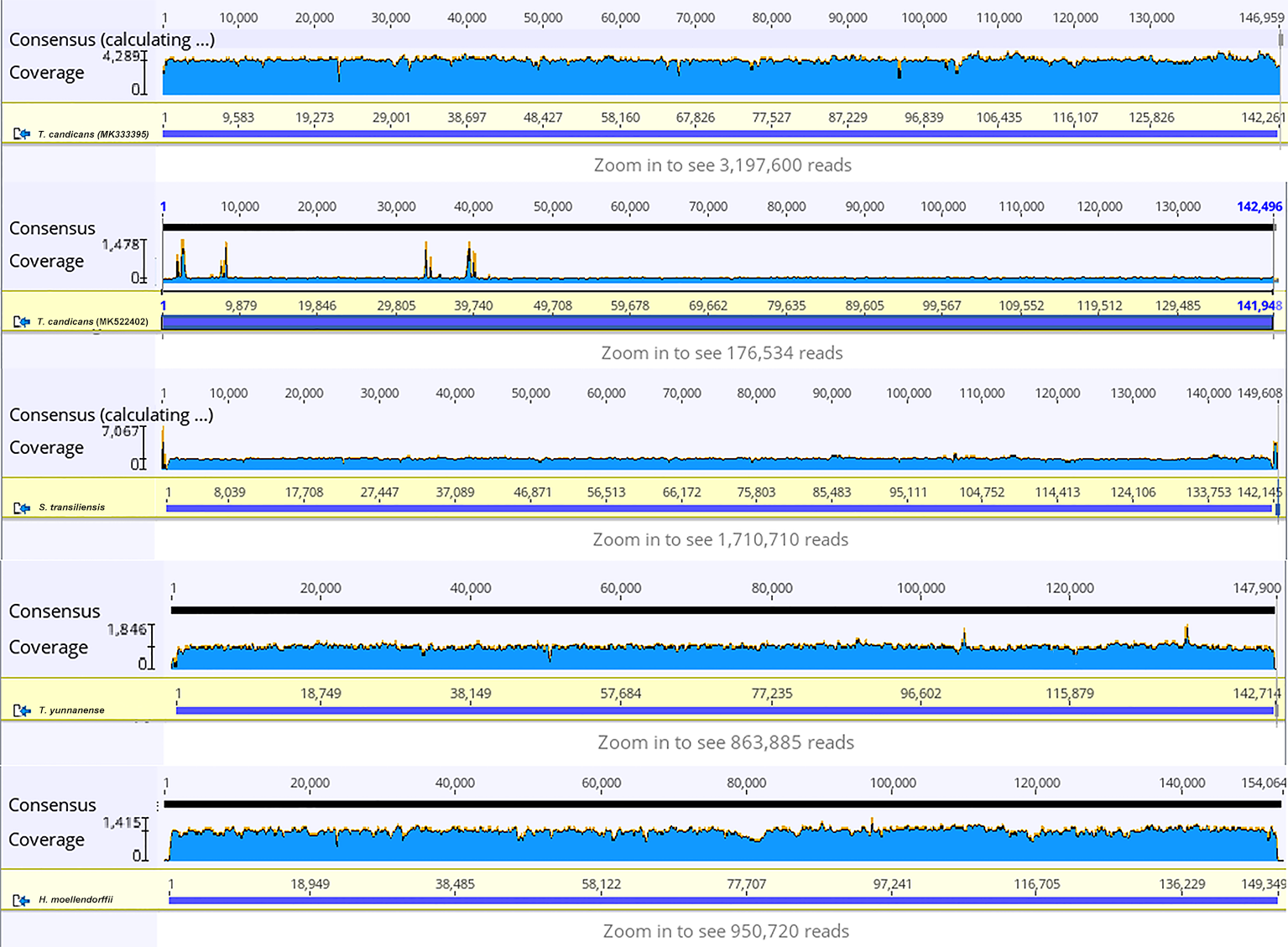

Supplement: Figure S1 — Blue belts represent reads that align to respective regions. Black belts represent chloroplast genomes, with numbers on it representing position on cp genomes. [file peerj-07-8063-s001.png]

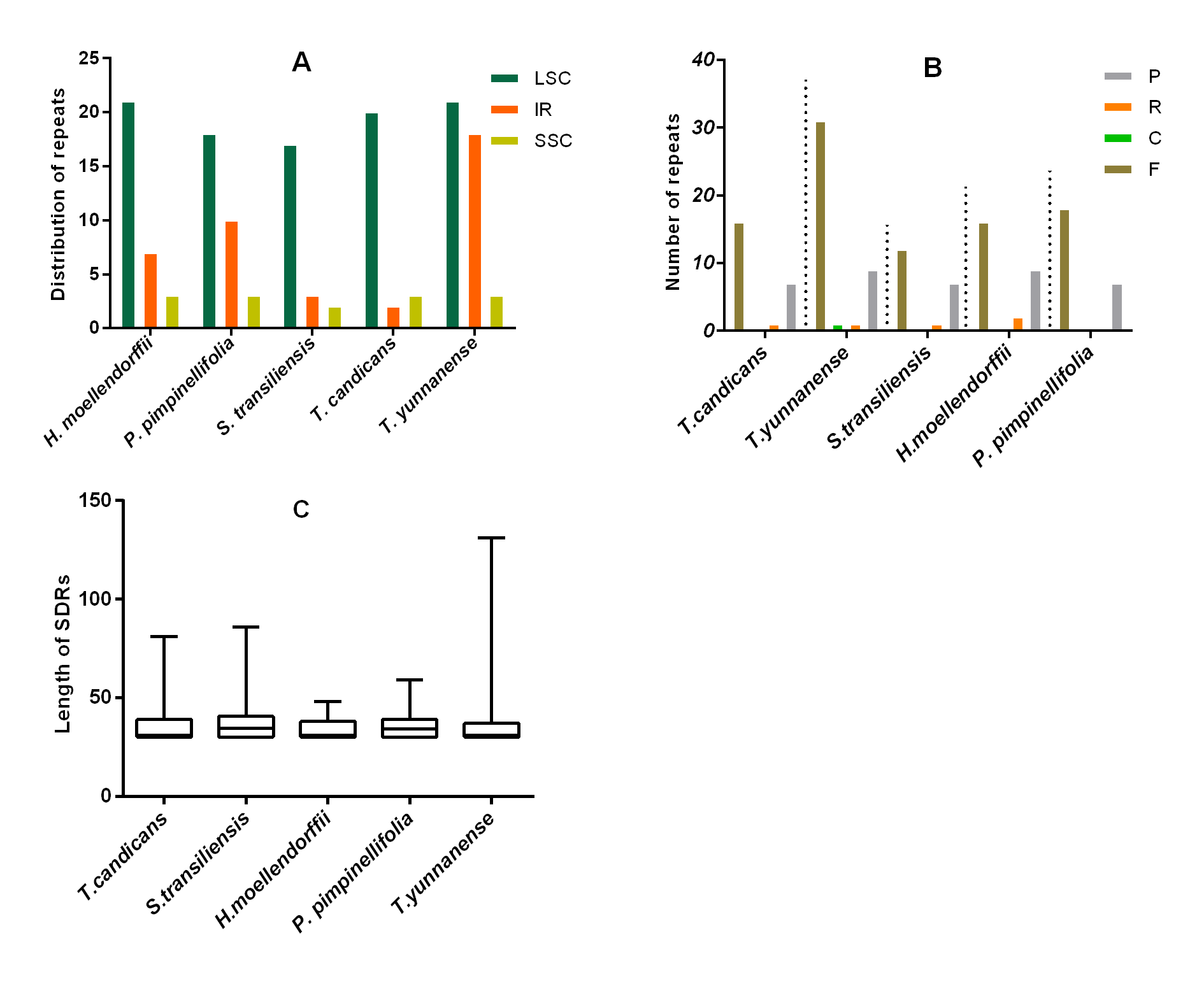

Supplement: Figure S2 — (A) Distribution areas of SDRs in the five cp genomes; (B) Categories of SDRs; (C) Length of SDRs. Abbreviation in Fig S2 (C):P represents palindromic SDRs, namely two SDRs are reverse complemented; C represents compound SDRs, namely SDRs that blend more than one form of SDRs; F represents direct SDRs, namely two SDRs in the same direction; R represents reverse SDRs, namely SDRs in adverse direction. [file peerj-07-8063-s002.png]

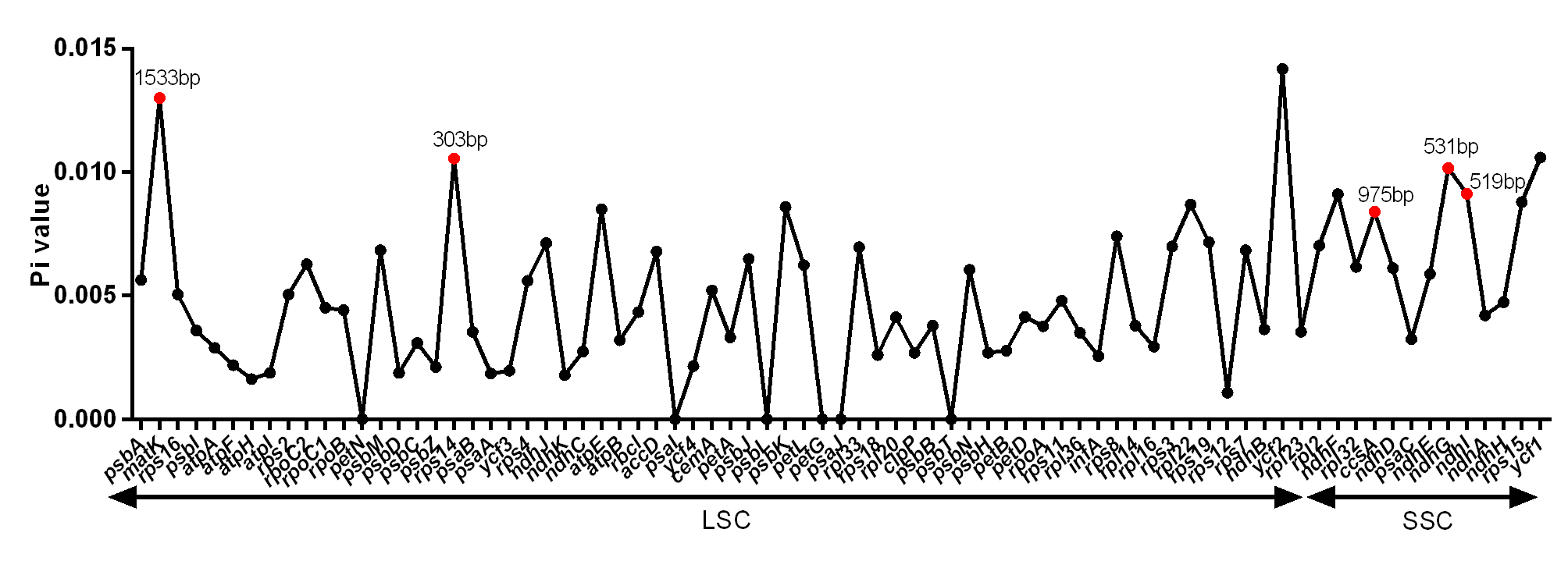

Supplement: Figure S3 — DNA segments that marked red dot and length are selected hyper-variable markers. [file peerj-07-8063-s003.png]
