# Supplementary material for: Sequencing and analyses on chloroplast genomes of Tetrataenium candicans and two allies give new insights on structural variants, DNA barcoding and phylogeny in Apiaceae subfamily Apioideae
Source: PeerJ. 2019 Nov 21;7:e8063. doi: 10.7717/peerj.8063 (PMC6875388; doi:10.7717/peerj.8063)
Supplement: Table S1 — DNA segments that marked red are applicable for candidate DNA barcodes to distinguish ’Danggui’ and its counterfeits. [file peerj-07-8063-s004.docx]

| Segments | Pi | Length | Number of | Number of | Segments | Pi | Length | Number of | Number of |
| --- | --- | --- | --- | --- | --- | --- | --- | --- | --- |
|  |  | (bp) | polymorpism sites | hapotypes |  |  | (bp) | polymorpism sites | hapotypes |
| *accD* | 0.00533 | 1473 | 23 | 7 | *rpl16 intron* | 0.00872 | 992 | 21 | 7 |
| *accD-ycf4* | 0.01477 | 972 | 35 | 7 | *rpl32-trnL* | 0.01676 | 1123 | 41 | 7 |
| *atpB-rbcL* | 0.0063 | 1097 | 19 | 6 | *rpl33-rps18* | 0.00885 | 803 | 18 | 6 |
| *atpF intron* | 0.00364 | 710 | 7 | 5 | *rpoB-trnC* | 0.01121 | 1225 | 35 | 7 |
| *atpF-atpH* | 0.01022 | 329 | 8 | 5 | *rpoC1 intron* | 0.01186 | 789 | 22 | 7 |
| *atpH-atpI* | 0.01357 | 1157 | 28 | 7 | *rpoC2* | 0.00494 | 4161 | 54 | 7 |
| *atpI-rps2* | 0.01356 | 345 | 8 | 5 | *rpoC2-rpoC1* | 0.0172 | 216 | 10 | 7 |
| *ccsA-ndhD* | 0.01501 | 1214 | 42 | 7 | *rps2-rpoC2* | 0.02286 | 237 | 12 | 6 |
| *cemA-petA* | 0.0078 | 936 | 19 | 6 | *rps3* | 0.00609 | 657 | 11 | 5 |
| *clpP intron1* | 0.00731 | 641 | 10 | 6 | *rps4-trnT* | 0.00916 | 377 | 8 | 6 |
| *clpP intron2* | 0.01314 | 869 | 28 | 7 | *rps8-rpl14* | 0.008 | 982 | 23 | 7 |
| *matK* | 0.01092 | 1518 | 46 | 7 | *rps12-trnV* | 0.01276 | 1827 | 64 | 5 |
| *ndhA intron* | 0.01194 | 1014 | 31 | 7 | *rps16 intron* | 0.0123 | 890 | 34 | 7 |
| *ndhC-trnV* | 0.01087 | 1078 | 31 | 7 | *rps16-trnQ* | 0.01571 | 1274 | 49 | 7 |
| *ndhD-ndhE* | 0.00924 | 1198 | 28 | 7 | *trnC-petN* | 0.00681 | 752 | 12 | 7 |
| *ndhF* | 0.00891 | 2222 | 54 | 7 | *trnE-trnT* | 0.03436 | 794 | 64 | 7 |
| *ndhF-rpl32* | 0.01988 | 1250 | 45 | 7 | *trnG intron* | 0.0109 | 794 | 23 | 7 |
| *ndhG-ndhI* | 0.0167 | 329 | 12 | 6 | *trnG-trnR* | 0.021 | 173 | 8 | 5 |
| *ndhH-ycf1* | 0.0076 | 1896 | 39 | 7 | *trnH-psbA* | 0.03549 | 192 | 15 | 7 |
| *petA-psbJ* | 0.03138 | 1091 | 64 | 7 | *trnK-rps16* | 0.02595 | 715 | 33 | 7 |
| *petB intron* | 0.01176 | 699 | 18 | 6 | *trnL-ndhJ* | 0.01017 | 853 | 21 | 7 |
| *petD intron* | 0.00776 | 957 | 18 | 7 | *trnQ-psbK* | 0.0118 | 349 | 10 | 5 |
| *petN-psbM* | 0.00723 | 1192 | 21 | 7 | *trnS-trnG* | 0.01161 | 564 | 18 | 7 |
| *psaA-ycf3* | 0.00564 | 716 | 9 | 5 | *trnT-psbD* | 0.01201 | 1493 | 42 | 7 |
| *psbA* | 0.00215 | 1183 | 7 | 6 | *trnT-trnL* | 0.01928 | 864 | 36 | 7 |
| *psbA-matK* | 0.01612 | 557 | 21 | 7 | *trnV-atpE* | 0.01446 | 479 | 18 | 7 |
| *psbB* | 0.00306 | 1527 | 13 | 6 | *ycf1* | 0.00976 | 5484 | 142 | 7 |
| *psbC-psbZ* | 0.00692 | 902 | 18 | 7 | *ycf2* | 0.01094 | 6378 | 178 | 7 |
| *psbE-petL* | 0.01458 | 1023 | 32 | 7 | *ycf2-trnL* | 0.00788 | 964 | 15 | 6 |
| *psbK-psbI* | 0.01619 | 273 | 9 | 7 | *ycf3 intron 2* | 0.00391 | 726 | 7 | 5 |
| *psbZ-rps14* | 0.01057 | 817 | 22 | 7 | *ycf3 intron1* | 0.00531 | 794 | 11 | 7 |
| *rbcL* | 0.00474 | 1428 | 16 | 7 | *ycf3-trnS* | 0.0076 | 944 | 18 | 7 |
| *rbcL-accD* | 0.00631 | 749 | 13 | 7 | *ycf4-cemA* | 0.16577 | 341 | 9 | 4 |
| *rpl2 intron* | 0.0077 | 832 | 17 | 7 |  |  |  |  |  |
