# Supplementary material for: Sequencing and analyses on chloroplast genomes of Tetrataenium candicans and two allies give new insights on structural variants, DNA barcoding and phylogeny in Apiaceae subfamily Apioideae
Source: PeerJ. 2019 Nov 21;7:e8063. doi: 10.7717/peerj.8063 (PMC6875388; doi:10.7717/peerj.8063)
Supplement: Table S2 [file peerj-07-8063-s005.docx]

| Sequence | Primer | | Length of | | | Number of | Number of | Nucleotide | Haplotype | Total | Private | Mismatch | | Fu and Li's | Tajima's | |
| --- | --- | --- | --- | --- | --- | --- | --- | --- | --- | --- | --- | --- | --- | --- | --- | --- |
|  |  |  | | | segment(bp) | Populations | individuals | diversity | diversity | haplotypes | haplotypes | analysis | | D* test | | D |
| *rpl16 intron* | F | 5'-GCTATGCTTAGTGTGCGACTCGTTG-3' | | 934 | | 4 | 10 | 0.00155 | 0.844 | 5 | 5 | unimodal | | -0.34 | | -0.04 |
|  | R | 5'-CCCTTCATTCTTCCTCTATGTTG-3' | | |  |  |  |  |  |  |  |  | | P > 0.10 | | P > 0.10 |
| *rps16 intron* | F | 5'-ATAGACGGCTCATTGGGA-3' | | | 769 | 37 | 37 | 0.00197 | 0.581 | 12 | 10 | unimodal | | -4.99 | | -2.57 |
|  | R | 5'-CGTGCGACTTGAAGGACA-3' | | |  |  |  |  |  |  |  |  | | P < 0.02 | | P < 0.001 |
| *trnQ-rps16* | F | 5'-CCCGGTATTCGGAGGTTCGA-3' | | | 1332 | 37 | 37 | 0.00172 | 0.763 | 16 | 12 | smooth curve | | -3.41 | | -2.31 |
|  | R | 5'-ATCGTGTCCTTCAAGTCGCA-3' | | |  |  |  |  |  |  |  |  | | P < 0.02 | | P < 0.01 |
| *trnL-trnT* | F | 5'-TGTTAGAACAGCTTCCATTGAGTCTC-3' | | | 898 | 36 | 36 | 0.00143 | 0.438 | 8 | 6 | smooth curve | | -3.46 | | -2.14 |
|  | R | 5'-CATTACAAATGCGATGCTCT-3' | | |  |  |  |  |  |  |  |  | | P < 0.02 | | P < 0.05 |
| *rpl32-trnl* | F | 5'-CAGTTCCAAAAAAACGTACTTC-3' | | | 1067 | 38 | 39 | 0.00197 | 0.758 | 15 | 10 | smooth curve | | -3.31 | | -1.60 |
|  | R | 5'-CTGCTTCCTAAGAGCAGCGT-3' | | |  |  |  |  |  |  |  |  | | P < 0.05 | | 0.10 > P > 0.05 |
| *rps16-trnk* | F | 5'-TTCCTTGAAAAGGGCGCTCA-3' | | | 545 | 38 | 52 | 0.00343 | 0.732 | 15 | 10 | unimodal | | -2.37 | | -1.30 |
|  | R | 5'-TACTCTACCGTTGAGTTAGC-3' | | |  |  |  |  |  |  |  |  | | 0.10 > P > 0.05 | | P>0.1 |
| *trnS-trnG* | F | 5'-AGAGAGGGATTCGAACCCTCG-3' | | | 412 | 38 | 38 | 0.00068 | 0.248 | 5 | 3 | smooth curve | | -2.14 | | -1.76 |
|  | R | 5'-GCGGGTATAGTTTAGTGGTAAAA-3' | | |  |  |  |  |  |  |  |  | 0.10 > P > 0.05 | | | 0.10 > P > 0.05 |
| *psbA-trnH* | F | 5'-GTTATGCACGAACGTAATGCTC-3' | | | 232 | 37 | 39 | 0.01224 | 0.631 | 8 | 4 | unimodal | | -0.16 | | 0.08 |
|  | R | 5'-CGCGCATGGTGGATTCACAATCC-3' | | |  |  |  |  |  |  |  |  | | P>0.1 | | P>0.1 |
| *TrnL-F* | F | 5'-CGAAATTGGTAGACGCTGCG-3' | | | 963 | 11 | 19 | 0.00098 | 0.468 | 6 | 5 | unimodal | | -1.78 | | -1.80 |
|  | R | 5'-ATTTGAACTGGTGACACGAG-3' | |  | |  |  |  |  |  |  |  | | P > 0.10 | 0.10 > P > 0.05 | |
