# Supplementary material for: Sequencing and analyses on chloroplast genomes of Tetrataenium candicans and two allies give new insights on structural variants, DNA barcoding and phylogeny in Apiaceae subfamily Apioideae
Source: PeerJ. 2019 Nov 21;7:e8063. doi: 10.7717/peerj.8063 (PMC6875388; doi:10.7717/peerj.8063)
Supplement: Table S3 [file peerj-07-8063-s006.docx]

| Country | Province | Location | Longitude (E) | Latitude (N) | Altitude (m) |
| --- | --- | --- | --- | --- | --- |
| China | Tibet | Cuona | 91°57'55" | 27°59'53" | 4380 |
| China | Tibet | Linzhi | 94°28'48" | 29°34'19" | 3076 |
| China | Tibet | Mangkang | 98°35'40" | 29°41'14" | 3869 |
| China | Tibet | Nielamu | 85°58'44" | 28°9'26" | 2300 |
| China | Tibet | Kangbu hot spring | 88°58'5" | 27°41'18" | 3851 |
| China | Tibet | Cuomei | 91°53'29" | 28°32'39" | 4412 |
| China | Tibet | Kangma | 89°40'54" | 28°33'20" | 4360 |
| China | Tibet | Nanmulin-Qiumu village | 89°1'8" | 29°37'30" | 4015 |
| China | Tibet | Nanmulin | 89°5'57" | 29°40'56" | 4002 |
| China | Tibet | Yadong | 88°54'26" | 27°29'5" | 3059 |
| China | Tibet | Zuogong | 97°37'59" | 29°53'28" | 4007 |
| China | Tibet | Bomi | 95°46'4" | 29°51'32" | 2727 |
| China | Tibet | Bomi-midui village | 96°29'40" | 29°30'4" | 3718 |
| China | Sichuan | Kangding | 102°4'18" | 30°4'36" | 2022-2652 |
| China | Sichuan | Kangding-Xinduqiao | 101°29'43" | 30°2'33" | 3461 |
| China | Tibet | Nielamu-Zhangmuqu village | 85°59'01" | 27°59'14" | 2220 |
| China | Sichuan | Batang | 99°6'38" | 30°0'17" | 2575 |
| China | Sichuan | Ganzi | 99°59'59" | 31°37'22" | 3390 |
| China | Tibet | Chaya | 97°16'50" | 30°41'19" | 3854 |
| China | Qinghai | Banma | 100°44'14" | 32°55'58" | 3524 |
| China | Tibet | Langxian-senmu village | 92°50'12" | 28°52'51" | 3771 |
| China | Sichuan | Litang | 100°16'11" | 29°59'46" | 4014 |
| China | Sichuan | Maerkang | 102°12'23" | 31°54'21" | 2647 |
| China | Tibet | Linzhou | 91°15'55" | 29°53'37" | 3755 |
| China | Tibet | Basu | 96°55'4" | 30°3'12" | 3288 |
| China | Tibet | Suoxian | 93°47'8" | 31°53'12" | 3989 |
| China | Sichuan | Daofu | 101°7'31" | 30°58'46" | 2979 |
| China | Sichuan | Muli | 101°23'1" | 28°30'22" | 3940 |
| China | Sichuan | Xiangcheng | 99°39'31" | 28°50'18" | 3300-3799 |
| China | Sichuan | Dege | 98°34'53" | 31°48'54" | 3304 |
| China | Sichuan | Songpan | 103°36'17" | 32°39'19" | 2865 |
| China | Sichuan | Xiaojin | 102°49'48" | 30°59'37" | 3340 |
| China | Sichuan | Wenchuan | 103°5'56" | 30°57'50" | 2220 |
| China | Tibet | Chayu | 97°26'39" | 28°39'25" | 3043 |
| China | Yunnan | Kunming | 102°38'7" | 24°57'31" | 2181 |
| China | Yunnan | Zhongdian | 100°8'18" | 27°22'46 | 3300 |
| China | Yunnan | Binchuan | 100°23'00" | 25°59'00' | 3003 |
| China | Yunnan | Lijiang | 100°12'53" | 27°0'50" | 2715 |
| China | Yunnan | Lanping | 99°28'22" | 26°27'19" | 3000 |
| China | Yunnan | Deqin | 98°54'42" | 28°29'10" | 3350 |
| China | Yunnan | Maanshan | 100°11'25" | 26°51'32" | 2386 |
| China | Sichuan | Meigu | 103°7'32" | 28°18'57" | 1820 |
| China | Sichuan | Huidong | 102°45'8" | 26°22'48" | 2880 |
